# Chronic low-grade inflammation and ovarian dysfunction in women with polycystic ovarian syndrome, endometriosis, and aging

**DOI:** 10.3389/fendo.2023.1324429

**Published:** 2023-12-13

**Authors:** Makoto Orisaka, Tetsuya Mizutani, Yumiko Miyazaki, Aya Shirafuji, Chiyo Tamamura, Masayuki Fujita, Hideaki Tsuyoshi, Yoshio Yoshida

**Affiliations:** ^1^ Department of Obstetrics and Gynecology, Faculty of Medical Sciences, University of Fukui, Fukui, Japan; ^2^ Department of Nursing, Faculty of Nursing and Welfare Sciences, Fukui Prefectural University, Fukui, Japan; ^3^ Department of Obstetrics and Gynecology, Ishikawa Prefectural Central Hospital, Ishikawa, Japan

**Keywords:** aging, endometriosis, follicular microenvironment, inflammation, ovarian dysfunction, polycystic ovarian syndrome

## Abstract

The ovarian microenvironment is critical for follicular development and oocyte maturation. Maternal conditions, including polycystic ovary syndrome (PCOS), endometriosis, and aging, may compromise the ovarian microenvironment, follicular development, and oocyte quality. Chronic low-grade inflammation can induce oxidative stress and tissue fibrosis in the ovary. In PCOS, endometriosis, and aging, pro-inflammatory cytokine levels are often elevated in follicular fluids. In women with obesity and PCOS, hyperandrogenemia and insulin resistance induce ovarian chronic low-grade inflammation, thereby disrupting follicular development by increasing oxidative stress. In endometriosis, ovarian endometrioma-derived iron overload can induce chronic inflammation and oxidative stress, leading to ovarian ferroptosis and fibrosis. In inflammatory aging (inflammaging), senescent cells may secrete senescence-associated secretory phenotype factors, causing chronic inflammation and oxidative stress in the ovary. Therefore, controlling chronic low-grade inflammation and fibrosis in the ovary would present a novel therapeutic strategy for improving the follicular microenvironment and minimizing ovarian dysfunction.

## 1 Introduction

Acute and chronic low-grade inflammation are two distinct types of inflammatory responses in the body. Acute inflammation is the immediate and transient response to tissue injury, infection, or foreign substances. Although acute inflammation can be associated with severe symptoms, it is a normal and essential process for eliminating harmful stimuli, initiating tissue repair, and restoring homeostasis (1, 2). Acute inflammation typically resolves within a few days or weeks.

In contrast, chronic low-grade inflammation is characterized by long-term, mild, systemic inflammation (3). Unlike acute inflammation, it may not manifest obvious signs or symptoms. Nevertheless, chronic low-grade inflammation disrupts immune function and causes tissue damage. It significantly contributes to the development and progression of various diseases, including cardiovascular diseases, metabolic disorders, neurodegenerative conditions, and certain types of cancer (4).

Although the relationship between inflammation and reproduction is complex, chronic low-grade inflammation can influence many ovarian reproductive processes (5, 6). Therefore, this review highlights recent findings regarding the impact of chronic low-grade inflammation on ovarian function.

## 2 Overview of chronic low-grade inflammation

Cytokines function as chemical messengers in the immune system, facilitating communication between cells and regulating immune responses. Various immune cells (e.g., macrophages, neutrophils, and lymphocytes) secrete cytokines, which have pro- or anti-inflammatory effects. Notably, pro-inflammatory cytokines are of particular significance in chronic low-grade inflammation. These cytokines, including tumor necrosis factor-alpha (TNFα), interleukin-1 beta (IL-1β), and interleukin-6 (IL-6), are released in response to inflammatory signals (7, 8). Moreover, the dysregulated production of pro-inflammatory cytokines promotes further inflammation and tissue damage via the inhibitor of nuclear factor kappa-B kinase subunit beta/nuclear factor kappa-B (NF-κB) and Janus kinase/signal transducer and activator of transcription signaling pathways.

Chronic low-grade inflammation and reactive oxygen species (ROS) are closely linked. ROS are chemically reactive oxygen-containing molecules, including superoxide (O_2_^-^), hydrogen peroxide (H_2_O_2_), and hydroxyl radicals (•HO). They play crucial roles in physiological processes, and a delicate balance with the antioxidant defense system maintains cellular homeostasis. However, excessive or uncontrolled ROS production causes oxidative stress in chronic low-grade inflammation (9). ROS can damage cellular components, including lipids, proteins, and DNA, and induce cell death. Consequently, oxidative stress activates several signaling pathways, contributing to further inflammation, tissue damage, and subsequent fibrosis (10, 11).

Fibrosis involves the excessive deposition of extracellular matrix (ECM) components, leading to tissue remodeling and scarring (12). Collagens are the main components of the ECM, and the increased deposition of type I and III collagen is a hallmark of fibrosis (13). Additionally, increased ROS levels and oxidative stress activate transforming growth factor-β1 (TGF-β1), which is a key cytokine involved in the fibrotic process (14). TGF-β1 promotes fibroblast proliferation, fibroblast-to-myofibroblast conversion, and ECM production and deposition (14, 15). Furthermore, fibrosis disrupts tissue architecture and can cause organ dysfunction in the liver, lungs, heart, kidneys, skin, and possibly ovaries (12, 16, 17).

## 3 Folliculogenesis in the ovarian cortex

The ovarian follicle, comprising an oocyte surrounded by somatic cells (cumulus cells [CCs], granulosa cells [GCs], and theca cells [TCs]), represents the basic functional unit for female reproduction. Folliculogenesis involves the activation of primordial follicles; continual growth through primary, secondary, preantral, and antral follicles; selection and maturation of dominant follicle(s); and ovulation. It is tightly regulated by pituitary gonadotropins (follicle‐stimulating hormone [FSH] and luteinizing hormone [LH]) and intraovarian regulators, including steroids, growth factors, and cytokines (18). More than 99% of follicles do not become dominant, leading to growth arrest and eventual atretic degeneration. Consequently, this results in the loss of follicles and oocytes (19). Oogenesis is a process involving the forming and development of a competent, mature oocyte within ovarian follicles. The surrounding CCs, GCs, and TCs provide essential steroids, growth factors, cytokines, and metabolites for oogenesis (20). Therefore, the follicular microenvironment is critical for oocyte growth, maturation, and the acquisition of developmental competence (21).

The ovarian stroma comprises both the cortex and medulla. The composition and organization of the stromal ECM change dynamically around the follicles (22). *In vitro* follicle culture experiments have demonstrated that growing follicles are sensitive to the stiffness of the surrounding ECM (16). The biomechanical pressure exerted by the surrounding stroma can influence follicular expansion and development through mechano-transduction pathways. For instance, Hippo signaling disruption and Akt stimulation are known to promote folliculogenesis in rodents and humans (22–24). Early follicles lack direct blood supply and receive various substances through passive diffusion from the surrounding stromal tissue (25). The progression of folliculogenesis beyond the preantral stage requires angiogenesis and vascularity in the ovarian stroma and TC layer to supply nutrients, oxygen, and gonadotropins.

## 4 Pro-inflammatory cytokines and follicular development

Altered levels of pro-inflammatory cytokines (e.g., TNFα, IL-1β, and IL-6) in the follicular microenvironment can negatively impact ovarian function (26, 27).

TNFα induced apoptosis and inhibited steroidogenesis in rat, bovine, and human GCs, indicating its negative impact on GCs (28, 29). In contrast, the effects of TNFα on TCs functions are inconclusive (30–32). Importantly, *TNFα* gene deletion in mice showed increased GC proliferation and decreased oocyte apoptosis, resulting in prolonged fertility (33). Therefore, excess TNFα is believed to adversely affect follicular development and cause follicular atresia (26). Moreover, TNFα can induce oxidative stress in porcine oocytes, causing DNA and mitochondrial damage and reducing oocyte quality (34).

IL-1β suppressed FSH and LH receptor (LHR) expression in mice, rat, and porcine GCs (26), as well as estradiol production in rodent and human GCs (26). Conversely, another study found that IL-1β stimulated bovine GC proliferation (35) and suppressed apoptosis in rat follicular cells (36). Notably, *IL-1* gene deficiency in mice resulted in an increase rather than a decrease in fertility (37). Therefore, IL-1β negatively impacts follicular development and oocyte maturation (38, 39).

IL-6 inhibited FSH-induced LHR expression in rat and porcine GCs (40, 41) and suppressed FSH-induced steroidogenesis in rat and bovine GCs (42, 43). Although a report indicated that IL-6 enhanced FSH-induced LHR expression in rat GCs (44), excess IL-6 is commonly associated with aging and believed to affect follicular development negatively (45–47).

Chronic low-grade inflammation can cause persistent oxidative stress in the ovary (48). Additionally, high ROS levels and low antioxidant capacity in follicular fluids were associated with poor pregnancy outcomes in human-assisted reproductive technology (ART) (49, 50). Maternal conditions, including polycystic ovary syndrome (PCOS), endometriosis, and aging, may compromise the ovarian microenvironment (47, 48, 51). Therefore, the following sections discuss whether and how chronic low-grade inflammation negatively impacts ovarian function in PCOS, endometriosis, and aging.

## 5 PCOS and chronic low-grade inflammation

PCOS is the most common cause of ovarian dysfunction, with a prevalence of 8–13% in reproductive-aged women (52). The clinical and pathological hallmarks of PCOS include oligo/anovulatory ovarian dysfunction, polycystic ovarian morphology, and clinical/biochemical hyperandrogenism (53). Follicular development frequently halts at the small antral stage in PCOS, preventing full maturation and ovulation. Hyperandrogenism, insulin resistance, hypothalamic-pituitary-ovarian axis imbalance (LH > FSH), and chronic low-grade inflammation are major contributors to the pathophysiological changes observed in PCOS (54, 55). In ART for PCOS, ovarian stimulation overcomes the follicular growth arrest and allows more oocytes to be retrieved. However, the percentage of high-quality oocytes/embryos was lower in PCOS cases than in non-PCOS cases (56).

**Figure 1:**
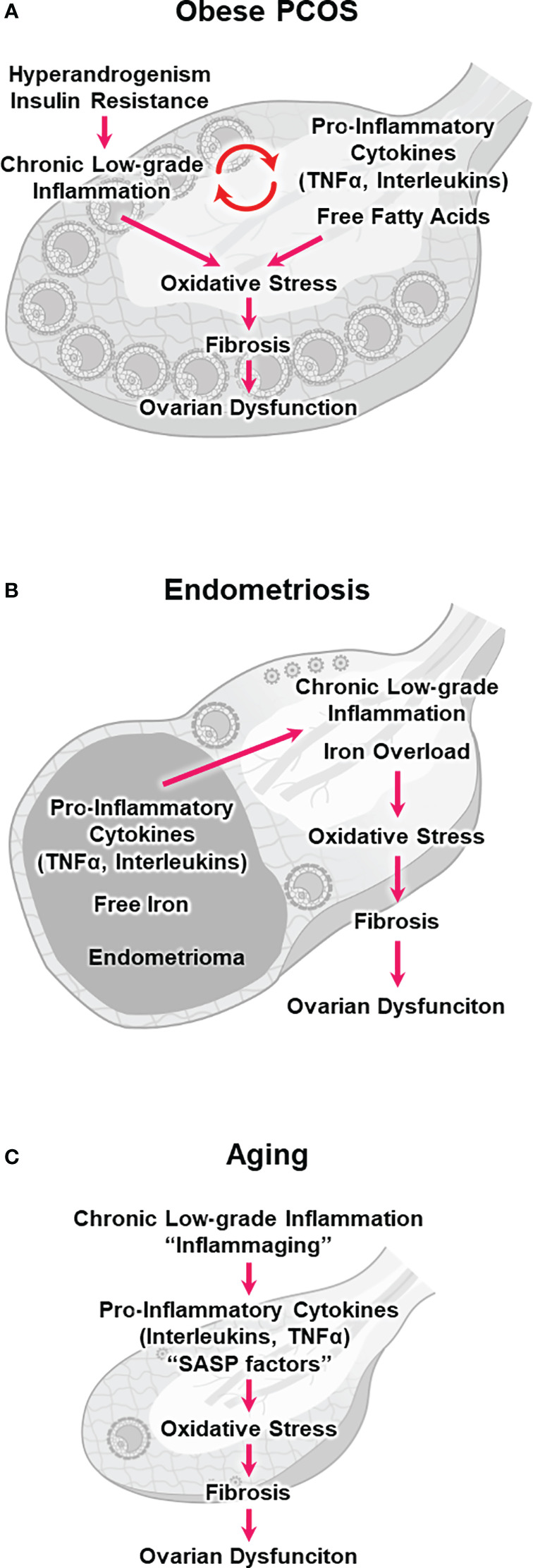
Hypothetical models illustrating chronic low-grade inflammation, oxidative stress, fibrosis, and ovarian dysfunction. **(A)** obesity and polycystic ovary syndrome (obese PCOS), **(B)** endometriosis, and **(C)** aging.

Follicular fluid is derived from blood and tissue fluid, and its composition correlates with that of the serum (27). Women with PCOS usually exhibit elevated serum levels of inflammatory markers, including C-reactive protein (CRP), TNFα, and IL-6 (6, 57). They also exhibit higher concentrations of TNF-α and interleukins in follicular fluids (58–60). Furthermore, macrophage and lymphocyte infiltration increases throughout the ovary in these women (61). Systemic and local chronic low-grade inflammation can increase oxidative stress in the ovary and negatively impact folliculogenesis in PCOS (6, 62) (**Figure 1A**). These phenomena are more pronounced in patients with obesity and PCOS (i.e., obese PCOS) than in normal-weight patients with PCOS (6).

The negative impact of obesity on ovarian function is evident, and ovulatory infertility prevalence in women with obesity is up to three times higher than that in those without obesity (63). Decreased pregnancy and birth rates in women with obesity were overcome by oocyte donation from those without obesity, suggesting that obesity disrupts folliculogenesis and reduces oocyte quality (64). Furthermore, excess adipose tissue produces inflammatory adipokines, including TNFα, IL-6, and free fatty acids (FFAs), which can contribute to cellular lipotoxicity, inflammation, and oxidative stress in the ovary (65). Oocytes from obese mice exhibited decreased germinal vesicle breakdown and polar body extrusion, along with abnormalities in spindle structure, chromosome alignment, and mitochondrial function (65–69). Therefore, these results suggest that obesity-induced oxidative stress negatively impacts both the meiotic and cytoplasmic maturation of oocytes.

Patients with obese PCOS who exhibit hyperandrogenism and insulin resistance are at a higher risk of abnormal folliculogenesis and poor oocyte competence (70). Hyperandrogenemia impairs the hypothalamic-pituitary-ovarian axis, resulting in a sustained increase in the gonadotropin-releasing hormone pulse frequency and the hypersecretion of LH over FSH (71). Hyperinsulinemia stimulates LH activity in TCs and promotes ovarian hyperandrogenism, which prevents follicular maturation and promotes follicular atresia (72). Additionally, insulin resistance can trigger a series of pro-inflammatory events, including hyperglycemia and oxidative stress (73, 74). Androgens promoted the differentiation of pre-adipocytes into mature adipocytes and increased lipolysis, resulting in an elevated release of FFAs (75, 76). Women with PCOS had higher FFA levels in follicular fluid, causing lipotoxicity and endoplasmic reticulum stress in the follicular microenvironment (77). These results suggest that hyperandrogenism and insulin resistance directly or indirectly stimulate chronic low-grade inflammation and increase ovarian oxidative stress (**Figure 1A**), which consequently compromises follicular maturation and oocyte quality in PCOS without follicle loss (57, 71).

Ovarian dysfunction in women with PCOS may also be correlated with ovarian fibrosis, which is characterized by excessive fibroblast proliferation and ECM deposition in the ovary (65, 78). Follicles in PCOS are surrounded by densely collagenized rigid stroma, which may inhibit follicular development (16). The molecular mechanisms underlying ovarian fibrosis in PCOS remain unclear. However, increased ROS levels and oxidative stress can activate TGF-β1, which is a key cytokine involved in tissue fibrosis (14). TGF-β1 levels were elevated in the serum and ovaries of women with PCOS and rat PCOS models (79–83). Therefore, these results indicate that ROS-induced TGF-β1 signaling may be involved in the pathophysiology of PCOS by stimulating ovarian fibrosis.

## 6 Endometriosis and chronic low-grade inflammation

Endometriosis is among the most common causes of infertility, with a prevalence of 10–15% in reproductive-aged women (84). It is a disease characterized by the growth of endometrial tissue outside the uterine cavity and estrogen-dependent chronic inflammation, primarily affecting pelvic tissues, including the ovary (i.e., endometrioma) (85). Although the effect of endometrioma on folliculogenesis remains inconclusive, follicular growth was significantly suppressed in the ovarian cortex surrounding the endometrioma (86). In ART, women with endometrioma had fewer retrieved oocytes than those without endometrioma, suggesting that endometrioma reduces follicular response to ovarian stimulation (84, 87, 88).

The endometrioma is surrounded by stroma and a single layer of columnar epithelial cells, but unlike other benign cysts, it is not surrounded by a capsule (17, 22). The toxic components of endometrioma, such as pro-inflammatory cytokines and ROS, can readily diffuse into the ovarian cortex and adjacent follicles (89). Indeed, altered levels of TNFα, interleukins, and ROS have been reported in the follicular fluid adjacent to the endometrioma (90, 91). These pro-inflammatory cytokines and ROS can induce oxidative stress and fibrosis in the ovary, impairing folliculogenesis and oocyte maturation (**Figure 1B**) (17, 92).

Free iron may also be harmful to the ovary, and the endometrioma fluid contains large amounts of free iron, approximately 10 times higher than the serum level (17). Iron levels were significantly elevated in the follicular fluid adjacent to endometriomas (93). Notably, iron is an essential mineral that plays a crucial role in various physiological processes, such as oxygen transport and energy production. However, ferrous iron (Fe^2+^) catalyzes the conversion from H_2_O_2_ to •HO via the Fenton reaction (Fe^2+^ + H_2_O_2_ → Fe^3+^ + •HO + OH^−^). •HO is among the most reactive and toxic ROS. Consequently, unbalanced and excessive iron levels (i.e., iron overload) can induce inflammation, oxidative stress, lipid peroxidation of the cellular membrane, and subsequent ferroptotic cell death (i.e., ferroptosis), a new type of programmed cell death (**Figure 1B**) (94–97). Although the effect of ferroptosis on ovarian function in endometriosis remains unclear, iron overload in the follicular fluid has been shown to induce GC ferroptosis and oocyte dysmaturation in endometriosis (98).

Ovarian dysfunction in endometriosis may also be correlated with ovarian fibrosis. One of the histologic features associated with endometriomas is fibrosis inside and outside the cyst (99). Indeed, follicular density was lower in the ovarian cortex adjacent to endometriomas, possibly because of the inhibition of angiogenesis, increased follicular atresia, and induction of fibrosis (100–102). Although the molecular mechanisms underlying ovarian fibrosis in endometriosis are yet to be elucidated, it is noteworthy that disruption in iron homeostasis induces organ fibrosis in the liver, heart, and pancreas (45). Endometrioma-derived free iron, pro-inflammatory cytokines, and ROS may cooperate with TGF-β1 signaling to promote stromal fibrosis around endometriomas (99, 103, 104).

Nevertheless, whether endometrioma reduces oocyte quality remains controversial. Many previous studies have shown poor ART outcomes in women with endometriosis (84, 105). Additionally, oocyte donation programs showed that embryos derived from women with endometrioma have lower implantation rates than those from women without endometrioma, suggesting reduced oocyte quality in endometriosis (91, 106). Oocytes from patients with endometrioma exhibited zona pellucida hardening, altered spindle structure, and decreased mitochondrial number (84, 107, 108). However, recent reports have shown that the presence of endometrioma does not affect clinical pregnancy and live birth rates, provided multiple oocytes are successfully retrieved and fertilized in the ART setting (87, 109). Although endometrioma negatively impacts the number of growing follicles and oocytes retrieved, whether the percentage of high-quality oocytes/embryos is lower in endometriosis remains inconclusive (84).

## 7 Aging and chronic low-grade inflammation

Female fertility begins to decline in the late 20s, and the decline accelerates rapidly beyond the mid-30s (110). Ovarian aging becomes noticeable in women in their late 30s and typically completes around the age of 50, suggesting that ovaries may be more susceptible to aging than other organs (111, 112). Notably, lower live birth rates and higher miscarriage rates in aged women were overcome by oocyte donation from young women, indicating that oocyte quality declines with age (113).

The progressive decline in oocyte quality and quantity is the main cause of age-related decline in female fertility. Multiple oocyte-related factors contribute to age-associated infertility, including chromosome mis-segregation, meiotic recombination errors, DNA damage, telomere shortening, mitochondrial dysfunction, genetic mutations, and protein metabolic dysregulation (111).

Additionally, the inflammatory follicular microenvironment and stromal fibrosis might contribute to ovarian aging (**Figure 1C**) (46, 47). Physiological aging is associated with chronic low-grade inflammation (114). Cellular senescence is the irreversible cell cycle arrest associated with aging. Senescent cells secrete inflammatory substances known as senescence-associated secretory phenotype (SASP) factors (e.g., IL-6, IL-1β, and IL-8). SASP factors induce chronic inflammation and oxidative stress in surrounding cells (47, 112). Inflammatory aging (i.e., inflammaging) has been implicated in age-related diseases and conditions, such as cardiovascular disease, neurodegenerative disorders, metabolic dysfunction, and possibly ovarian dysfunction (112).

Serum concentrations of IL-6, IL-1β, and TNFα were modestly but definitely increased in aged women (115). Aged women also showed higher IL-6 concentrations and lower antioxidant levels in follicular fluids (27, 116). Aged mice exhibited increased macrophage infiltration and fusion and pro-inflammatory cytokine expression in the ovary (45, 46, 117). Furthermore, the NF-κB pathway, which is a major inflammatory signaling pathway, was also activated in the ovaries of aged mice (118).

Whether and how inflammaging affects physiological ovarian aging remains unknown. Nevertheless, sustained small changes in pro-inflammatory cytokine expression and subsequent long-term accumulation of oxidative stress may deteriorate the follicular microenvironment and cause GC apoptosis and follicular atresia (**Figure 1C**) (46, 47, 119). Cellular senescence and SASP secretion were significantly increased in the ovaries of aged mice, particularly in TCs (120). Oocyte-somatic cell communication is indispensable for folliculogenesis and oocyte maturation (18, 121, 122). Loss of support from surrounding CCs, GCs, and TCs can result in defects in oocyte chromosomal, genetic, mitochondrial, and cytoplasmic factors, compromising oocyte quality (111, 112).

Furthermore, ovarian ECM deposition and stromal fibrosis were also observed in aged mice and humans (16, 45, 123). Transcriptome analysis of aged women showed that hypoxia stress response-associated genes (e.g., genes downstream of the hypoxia-inducible factor-1 pathway) were overexpressed in CCs, suggesting a hypoxic microenvironment in aging follicles (124). In the aging ovary, stromal changes, such as increased fibrosis and decreased angiogenesis, may induce a stressed environment (e.g., hypoxia), leading to impaired folliculogenesis and reduced oocyte quality (47).

## 8 Conclusion

Recent research suggests that chronic low-grade inflammation is associated with ovarian dysfunction in women with PCOS, endometriosis, and aging. However, our understanding of the impact of the pro-inflammatory microenvironment and fibrotic ECM remodeling on folliculogenesis and oocyte maturation remains limited. Therefore, explaining the pathologies of ovarian dysfunction in PCOS, endometriosis, and aging by chronic low-grade inflammation is still difficult. It remains unclear whether chronic low-grade inflammation and fibrosis are the cause or consequence of these conditions. Nevertheless, controlling chronic low-grade inflammation and fibrosis in the ovary would represent a novel therapeutic strategy to improve the follicular microenvironment. Animal models have indicated that antioxidants (27, 51), insulin-sensitizing drugs (125), anti-aging drugs (senolytics) (126), immune checkpoint inhibitors (127), and antifibrosis drugs (125) may help minimize follicular depletion and oocyte quality decline. However, further studies are required to determine whether these drugs overcome ovarian dysfunction in PCOS, endometriosis, and aging.

## Author contributions

MO: Conceptualization, Writing – original draft. TM: Resources, Visualization, Writing – review & editing. YM: Resources, Visualization, Writing – review & editing. AS: Resources, Visualization, Writing – review & editing. CT: Resources, Visualization, Writing – review & editing. MF: Resources, Visualization, Writing – review & editing. HT: Resources, Visualization, Writing – review & editing. YY: Supervision, Writing – review & editing.
